# Editorial: Robust machine learning

**DOI:** 10.3389/frai.2026.1811728

**Published:** 2026-03-17

**Authors:** Yuexuan An, Xingyu Zhao, Mingjing Du

**Affiliations:** 1 College of Computer Science and Software Engineering, Hohai University, Nanjing, China; 2 Key Laboratory of New Generation Artificial Intelligence Technology & Its Interdisciplinary Applications (Southeast University), Ministry of Education, Nanjing, China; 3 College of Computer Science and Technology, Nanjing University of Aeronautics and Astronautics, Nanjing, China; 4 School of Artificial Intelligence and Computer Science, Jiangsu Normal University, Xuzhou, China

**Keywords:** deep learning, machine learning, machine learning systems, open-world machine learning, robust machine learning

Machine learning (ML) has become a key technology in computer vision, autonomous driving, robotics and many other domains. As ML systems are deployed in complex and dynamic environments, robustness, reliability and adaptability emerge as core requirements. Models must remain effective when data are noisy or corrupted, when distribution shifts occur across datasets, devices or time, and when they are exposed to open-world scenarios (Kejriwal et al., 2024) where novel or even adversarial inputs appear. At the same time, the field faces questions about data efficiency (An et al., 2023), label ambiguity (An et al., 2025), and interpretability (Hernán et al., 2025), all of which affect how reliably systems can be understood and monitored in practice.

This Research Topic, *Robust machine learning*, was launched to address these challenges. Its aim is to bring together work that advances resilient ML systems through improved generalization and transfer learning, robustness in open-world conditions, better data quality and preprocessing, data-efficient utilization, and more interpretable models. The four contributions accepted in this Research Topic cover diverse application areas yet share a common goal: to study how robustness can be defined, measured and improved when models interact with real data and real constraints.

In Alrusaini, the author investigate a security-relevant task where images are routinely resized, compressed, cropped or contaminated by noise before analysis. They systematically compare several deep steganalysis architectures under such transformations and report how detection performance changes relative to clean images. The study shows that models which appear strong on clean data can differ markedly in robustness once realistic perturbations are introduced. Conceptually, this work illustrates the importance of aligning robustness evaluation with real-world perturbations and contributes directly to the Research Topic's focus on robustness under open-world and practically motivated image changes.

The article Sirri and Guyeux, examines robustness from the perspective of feature construction in a time-varying environment. The authors compare two types of meteorological risk indicators for predicting firefighter interventions: one derived from traditional meteorological products and one generated by large language models. By evaluating multiple learning algorithms across different activity levels and seasons, they show that AI-generated risks can improve predictive performance in many regimes while also identifying situations where the traditional pipeline remains competitive.

This contribution highlights that robustness is not only about model architecture; it also depends on data quality, preprocessing and the stability of feature pipelines under shifting conditions.

In Guo et al., robustness is studied in a clinical context characterized by heterogeneous devices and acquisition protocols. An AI model estimates bone mineral density from routine CT scans and is compared against quantitative CT as a reference standard. Crucially, the authors assess performance across CT scanners from multiple vendors and analyze inter-device consistency, rather than relying on a single acquisition setting. This work anchors the Research Topic's interest in generalization and transfer learning, showing how robustness in medical AI must be evaluated with respect to device and domain variation if models are to be trusted and adopted in real-world healthcare workflows.

The article, Fang et al., addresses robustness through data-efficient and data-centric design. Working with educational data that are high dimensional and potentially noisy, the authors propose an optimizer-based feature selection procedure to identify a compact set of informative variables, combined with an ensemble prediction framework that integrates optimized base learners. By focusing on a smaller, more stable feature subset while maintaining strong predictive performance, the study demonstrates how robustness can be improved by reducing redundancy and emphasizing data-efficient learning. This contribution illustrates the Research Topic's themes of robust representation and efficient utilization of limited or imperfect data.

These four articles advance robust machine learning along complementary dimensions. They emphasize that robustness should be defined against concrete challenges and variability, including real-world transformations, shifts in feature-generation pipelines, device heterogeneity, and high-dimensional noise. They show that improvements can come from different points in the pipeline: architecture and training, feature validation, cross-device evaluation, and data-centric strategies for stability and efficiency.

These contributions highlight promising directions for future work, including robust representation learning that transfers across domains and devices, training strategies that remain stable under distribution shift, open-world techniques for detecting and handling out-of-distribution or anomalous inputs, and interpretable models that help practitioners diagnose failures. Linked to real-world security, emergency response, healthcare, and education, these directions reinforce robustness as a guiding principle for designing, evaluating, and deploying modern ML systems in practice.

We hope this Research Topic will encourage research at the intersection of robust modeling, data quality, open-world reasoning, and interpretability, and will support the development of machine learning systems that can be trusted to perform reliably in increasingly complex real-world deployments.
